# Transcriptional and epigenetic regulation of T-helper lineage specification

**DOI:** 10.1111/imr.12204

**Published:** 2014-08-14

**Authors:** Subhash K Tripathi, Riitta Lahesmaa

**Affiliations:** 1 Turku Centre for Biotechnology, University of Turku and Åbo Akademi UniversityTurku, Finland; 2 National Doctoral Programme in Informational and Structural BiologyTurku, Finland; 3 Turku Doctoral Programme of Molecular Medicine (TuDMM), University of TurkuTurku, Finland

**Keywords:** T-helper cell, gene regulation, transcription factors, STATs, epigenetic modification, SNPs

## Abstract

Combined with TCR stimuli, extracellular cytokine signals initiate the differentiation of naive CD4^+^ T cells into specialized effector T-helper (Th) and regulatory T (Treg) cell subsets. The lineage specification and commitment process occurs through the combinatorial action of multiple transcription factors (TFs) and epigenetic mechanisms that drive lineage-specific gene expression programs. In this article, we review recent studies on the transcriptional and epigenetic regulation of distinct Th cell lineages. Moreover, we review current study linking immune disease-associated single-nucleotide polymorphisms with distal regulatory elements and their potential role in the disease etiology.

This article is part of series of reviews covering Transcriptional and Epigenetic Networks Orchestrating Immune Cell Development and Function appearing in Volume 261 of *Immunological Reviews*.

## Introduction

CD4^+^ T lymphocytes are the key players of adaptive immune system. Depending on the nature of antigen signal and type of cytokines produced by antigen-presenting cells (APCs), naive CD4^+^ T cells differentiate into an array of functionally different effector T-helper (Th) and regulatory T (Treg) cell subsets 1–3. Precise control of cellular specification and commitment during differentiation of these effector Th and Treg cells is critical for immune regulation and the immune protection against various infections. However, uncontrolled regulation of differentiation program can result in the pathogenesis of various autoimmune and allergic diseases.

Molecular mechanisms of CD4^+^ T-cell specification into distinct subsets involves extrinsic and intrinsic factors that mediate changes in the gene expression programs to define the fate of specific Th-cell subset while opposing the other subsets 4–6. Transcription factors (TFs) are vital players in priming the transcription of lineage-specific genes that drive the differentiation potential toward a particular lineage while restricting alternative fates. In the past decade, much has been learned about the role of TFs driving lineage commitment during CD4^+^ T-cell differentiation 7–9. In the following sections, we discuss the role of TFs in regulating the Th-cell differentiation and how integrated networks of these TFs co-expressed in a cell are driving specification and commitment of given Th-cell phenotypes. In addition, we point out the role of various epigenetic mechanisms in controlling the expression of lineage-specific genes important for determining the fate of specific cell lineage 4,7,10,11.

Several excellent review articles have discussed this theme earlier 5–7,12,13. In this review, we focus on the recent studies on transcriptional and epigenetic processes that aim at understanding the global ‘transcriptional’ and ‘chromatin landscape’ in guiding cellular specification and commitment during Th-cell differentiation. We also discuss studies proposing how disease-associated single-nucleotide polymorphisms (SNPs) may regulate the structure and function of *cis*-elements that determine lineage-specific gene expression programs during specification and commitment of differentiating Th cells.

## Regulation of gene expression

In multicellular organisms, the process of cellular specification and commitment during development of a cell lineage is directed by overlapping network of gene regulatory mechanisms that mediate spatiotemporal changes in the gene expression program that results in various cell fates 14,15. The network of TFs and epigenetic mechanisms are instrumental in governing gene regulatory programs that drive specific changes in the gene expression programs. Most of the TFs contain two structural domains: first, DNA-binding domain that specifically recognizes and binds to the specific DNA sequence, and second, trans-activation domain (protein interaction domain) that enables the recruitment of other TFs or regulatory proteins, chromatin remodeling complexes, and histone-modifying enzymes that co-modulate the gene expression programs. TFs that function as pioneer factors participate in creating DNase1 hypersensitive sites (DHS) that represent open chromatin structures within nucleosome-free regions harboring regulatory *cis*-elements 16,17. Distinct TFs bind to their regulatory *cis*-elements within the nucleosome-free regions determined by DHSs 18,19. Further, the improvement of the high-throughput sequencing and array technologies have enabled to construct global maps of a given TF binding in the genome and the correlation of such binding with global gene expression profiles or ‘transcriptomes’. The impact of a TF for a specific cellular transcriptome can be determined by identifying its global targets by coupling RNA interference (RNAi) with chromatin immunoprecipitation (ChIP) techniques followed by either microarray or high-throughput sequencing technologies 7,20,21. Thus, genome-wide mapping of TF-binding sites has identified thousands of sequence-specific DNA sites in the genome for TFs through which they regulate the expression of their target genes in many cell types in response to environmental cues promoting the differentiation and development. Cell fate decisions during lineage specification cannot be determined by a single TF alone, but a highly coordinated network of a series of TFs is required to govern the functional gene expression programs in the cells 22,23. Notably, integrative analysis of SNPs from various genome-wide association studies (GWAS) databases revealed that several disease-associated SNPs were enriched within TF-binding motifs suggesting that the disruption of TF-binding sites by SNPs can lead to changes in the expression profile of TF target genes 11,23,24.

**Fig 1:**
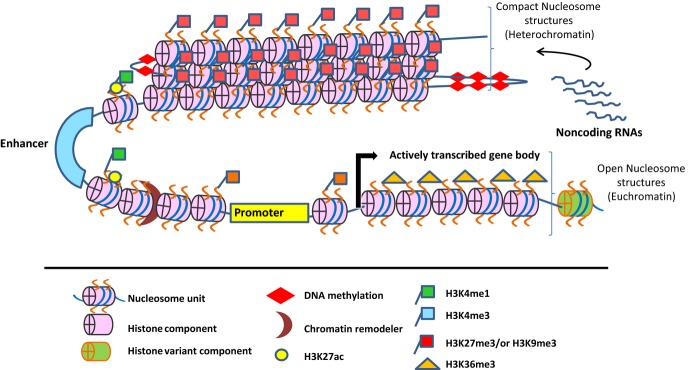
Epigenetic mechanisms in the regulation of gene expression. Epigenetic mechanisms maintain chromatin structure either in a transcriptionally silent ‘heterochromatin’ or open and active ‘euchromatin’ states. Both heterochromatin and euchromatin structures are marked with distinct epigenetic modifications such as DNA methylation and or histone modification. Non-coding RNAs also contribute to epigenetic regulation of gene expression.

Epigenetic mechanisms also regulate gene expression programs. Epigenetic factors control the accessibility of TFs to their cognate *cis*-regulatory regions within the highly ordered chromatin structure 14,25–27. Specific TFs recruit chromatin remodeling complexes to specific regulatory regions which then participate in gene regulation through their enzymatic or nucleosome remodeling activities 28,29. DNA methylation, posttranslational modification of histone tails, chromatin remodeling complexes, chromatin interaction/chromosome confirmation, and non-coding RNAs (ncRNAs) are the major epigenetic factors that participate in the gene regulation by either activating or repressing gene expression programs 29–32. The epigenetic modifications associated with active chromatin state open the tightly packed chromatin structure and expose *cis*-regulatory elements available for the binding of TFs and other regulatory DNA-binding proteins to activate gene expression and vice versa for the epigenetic modifications associated with repressive chromatin structure (*Fig. *1). It has been shown that these epigenetic modifications are generated and erased in a precise fashion during the course of differentiation and development of various cells and tissues, and are altered in response to intrinsic and extrinsic stimulus.

## *Cis*-regulatory DNA elements

*Cis*-regulatory DNA elements located on different regions of the genome, such as promoters, enhancers, silencers, insulators, and locus control regions (LCRs) serve as sites for epigenetic modifications and participate in the regulation of gene expression by regulating these sites 33. The *cis*-regulatory sites are highly conserved among vertebrates and contain hubs for the binding of multiple TFs 34. These *cis*-regulatory modules can be present several kilo bases away from the gene body and still regulate gene expression through distinct mechanisms including long range interactions and DNA looping 35,36.

## Chromatin remodeling and chromosomal rearrangement, interaction, and conformation

Chromosomes are made of bundles of chromatin structures composed of the basic repeating structural unit of chromatin: the nucleosome. The nucleosomes are packaged into the complex higher order chromatin structure that controls the accessibility of regulatory proteins to *cis*-regulatory elements in the genome that regulate gene expression 37. However, chromatin structure can undergo dynamic changes (chromatin remodeling or nucleosome positioning) that cause dissolution of histone octamers allowing transcription regulatory factors and basal transcription machinery to access open DNA template for gene transcription 14,25–27.

Several studies have shown that chromatin remodeling causes positioning of nucleosomes resulting in transcriptionally active sites (nucleosome-free regions) flanked by two nucleosomes 26,38. In addition, chromatin or nucleosome remodeling regulates chromatin structure and nucleosome dynamics through an ATP-dependent process 26. Several enzymatic factors that regulate histone–DNA interactions during chromatin remodeling process are referred to as chromatin or nucleosome remodelers. The chromatin remodelers form complexes of several proteins that utilize energy generated due to ATP hydrolysis to slide or dissolve histone octamers leading to remodeling of a nucleosome. Nucleosome remodelers such as SWI/SNF, ISWI, SWR, Mi-2/CHD, and INO80 are involved in the regulation of gene transcription 39–41. During the regulation of specific target gene transcription, these remodeling complexes are recruited by TFs to target genes 42–44.

## Histone modification

Posttranslational modification of the histone proteins is a key mechanism of epigenetic regulation of gene expression. The major posttranslational modifications associated with histone tails are methylation, acetylation, phosphorylation, ubiquitylation, and sumoylation. Depending on the type of posttranscriptional modifications, their location in the genome and their combinatorial patterns, histone modifications are associated with either active or repressive chromatin states. The regulatory proteins that participate in the modification of histone proteins resulting in the active and silent chromatin state are the group of trithorax group (TrxG) and polycomb group (PcG) protein complexes, respectively 27. Furthermore, combinatorial and comparative analysis on global profiles of histone modifications has defined three discrete chromatin states: active, poised, and silent 45–48. For instance, mono/di/tri-methylation status at the lysine 4 (K4me1/me2/me3) residue of histone 3 (H3) is associated with permissive gene expression. On the other hand, tri-methylation at the lysine 9 and 27 (H3K9me3, and H3K27me) residues of H3 protein is associated with repressive gene expression. Furthermore, co-localization of H3K4me3 and H3K27me3 on a given promoter is referred to as ‘bivalent domain’ or ‘bivalent chromatin state’ that has been associated with poised chromatin states in different cell types 49. H3K36me3 modification is spread along actively transcribed gene body 50. Histone acetylation is critical for chromatin function and associated with active gene expression. Acetylation of histone tails is governed by two key enzymes with opposite function, histone acetyltransferases (HATs) that add acetyl group on the histone tails and histone deacetylases (HDACs) that remove acetyl group from the histone tails. Notably, several studies indicate that acetylation of lysine 27 residue of H3 histone is associated with active chromatin domains and permissive transcription 51,52. Therefore, depending on the genomic location and type of posttranslational modification, histone modifications serve key functions in the gene regulation during development and differentiation of cell lineages 45,46,49,52.

## DNA methylation

In higher organisms, DNA methylation is the key component of epigenetic regulation of gene expression. Methylation of cytosine residues of CpG dinucleotides specifically at promoter regions of genes mediate silencing of gene transcription by limiting the accessibility of the TFs to the relevant target DNA 53–55. In somatic cells, DNA methylation is believed to be the most stable epigenetic mark with epigenetic memory as it is maintained in succeeding generations by DNMT1 (DNA methyltransferase 1), a member of DNA methyl transferase enzyme family. Both in plants and higher organisms, characterization of DNA methylome using genome-wide methylation profiles revealed methylation of DNA both at the CpG and non-CpG sites that are associated with promoters and actively transcribed gene body, respectively 55–57. Furthermore, global mapping of DNA methylation profiles both in induced pluripotent stem (iPS) cells and embryonic stem (ES) cells have revealed differences in DNA methylation profiles between these two cell types, that questions the efficacy of iPS cells as an alternative to ES cells for cellular reprogramming 58,59. In addition, though the role of DNMTs in DNA methylation is well studied, the molecular mechanisms and signaling events by which DNMTs participate in DNA methylation program are not well understood 60,61. Recently TET proteins were discovered as new regulators of DNA methylation and there are excellent current reviews on them 62–66. Interestingly, 5-hydroxymethylation (5hmC) of cytosine, a modification mediated by TET proteins has been shown to be deposited in actively transcribed gene regions as well as over a subset of enhancer elements and to be potentially regulated in pluripotency as well during differentiation of hematopoietic stem (HSc) and of ES cells 67–70.

## ncRNAs

Aside from DNA methylation and histone modification, ncRNAs play an important role in epigenetic regulation of gene expression. Depending on their transcript size, ncRNAs are classified into two major catagories: more than 200 nucleotides as long ncRNAs and less than 200 nucleotides as small ncRNAs 71. Further, small ncRNAs can be categorized into small interfering RNAs (siRNAs), microRNAs (miRNAs), PIWI-interacting RNAs (piRNAs), and small nucleolar RNAs (snRNAs). Each of these ncRNAs utilizes distinct mechanisms to regulate gene expression 72. For instance, miRNAs regulate gene expression by binding to the coding or untranslated regions (UTRs) of target mRNA transcripts and resulting in either mRNA degradation or inhibition of translation. LncRNAs show high degree of tissue and species-specific expression and there are reports on their role in gene regulation 73. Among the lncRNAs, long intergenic ncRNAs (lincRNA) are widely studied 74. Genome-wide analyses of lincRNAs in cellular differentiation systems (including T cells) have revealed cell-specific lincRNAs 75.

## Transcriptional control of Th lineage specification and commitment

**Fig 2:**
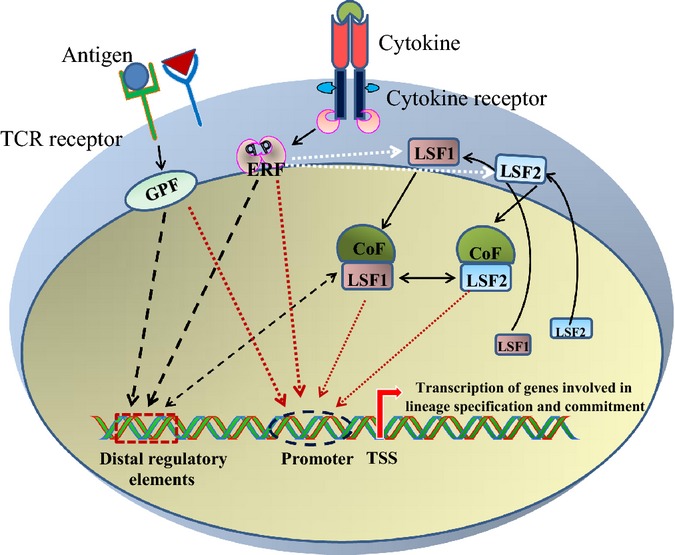
Role of transcription factors in inducing the transcription of lineage-specific genes in T cells. Antigens and cytokines are extracellular signals received by T cells through T-cell receptors (TCRs) and cytokine receptors. Ligation of antigen to TCRs activate general acting pioneer transcription factors (GPFs), such as NF-κB, NFAT, and AP-1 and cytokines binding to cognate cytokine receptors lead to activation of environment response factors (ERFs), such as STATs. These pioneer transcription factors independently or synergistically regulate global chromatin state and the expression of a lineage-specifying factor (LSF). LSFs and other co-factors further bind to the pre-existing chromatin landscape created by pioneer factors to regulate transcription of genes involved in lineage-specific gene expression program.

We and others 76–79 have shown that the process of lineage specification and commitment of Th-cell lineages is governed by their unique gene expression patterns. Each Th-cell lineage can be distinguished on the basis of their unique transcriptional profiles. The lineage-specific transcriptional profiles are guided by the TFs that determine the fate of a specific T-cell lineage 23,80–84 (*Fig. *2). TFs coordinate lineage-specific gene expression program by dictating the transcription of cell-specific genes that determine the fate of a given Th-cell lineage and limit the development of other Th-cell lineages 6,7,85. Several studies have documented the role of various TFs in controlling the lineage specification and commitment program during the different stages of T-cell development 86,87.

## TCR-stimulated general pioneer factors and cytokine environment sensor STATs

**Fig 3:**
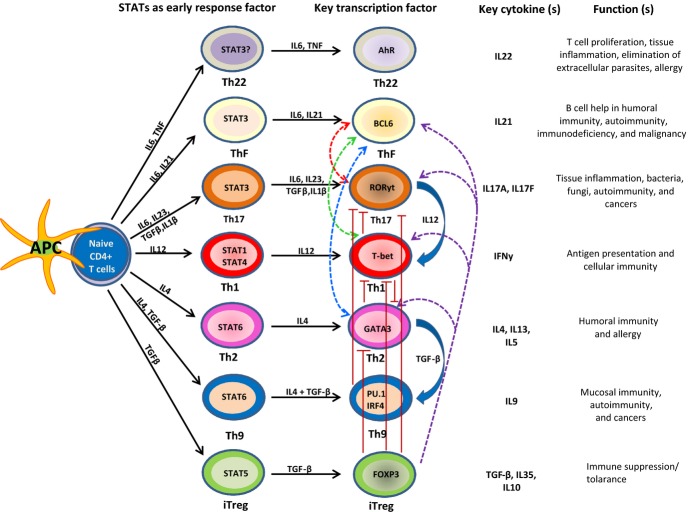
CD4^+^ T-helper cell subsets and their plasticity. Depending on the nature of antigen stimulation signal received from APCs, naive CD4^+^ T cells differentiate into distinct effector T-helper and regulatory T-cell subsets, which are characterized by expression of lineage-specific transcriptional regulators, cell surface markers, and secretion of key cytokines. Due to plasticity, differentiated effector CD4^+^ T cells can convert from one cell type into another. Uncontrolled regulation of lineage specification and commitment can result in development of inflammatory and allergic diseases.

The first determining factor that contributes to the CD4^+^ T-cell specification into a specialized Th or Treg cell is TCR stimulation. Strong TCR signal favors Th1, Th17, and Tfh cell differentiation, while weak TCR signal promotes development of Th2 cells and induced Treg cells (iTreg) 88–91. TCR-induced pioneer factors work in coordination to pass TCR-induced signal which together with other signals participate in CD4^+^ T-cell specification. However, how TCR-derived pioneer factors dictate differentiation of various subtypes is not fully understood. In addition to the TCR-induced pioneer factors, cytokine environment that stimulated T cell is an important factor for determining the fate of a specific T-cell lineage. The cytokines secreted by APCs mainly signal through Type I/II cytokine receptor superfamily that uses Janus kinase–signal transducer and activator of transcription (JAK–STAT) signaling pathway to convert cytokine-guided environmental signals into intrinsic signals that initiate specific gene expression program 92. STATs are DNA-binding regulatory proteins which upon cytokine stimulation function as TFs to drive selective gene expression program that determines specification of a relevant Th-cell subset. There are seven members in the STAT family (STAT1-4, 5 a & b, and 6) which are expressed in different Th-cell subsets and drive their differentiation upon stimulation with a given cytokine 93,94. Notably, there may be multiple cytokines that activate number of STATs involved in the initiation of differentiation of a given Th-cell subset 12. For example, STAT1 and STAT4 become activated in Th1 cells, STAT6 in Th2 and Th9 cells, STAT3 in Th17 and Tfh cells, and STAT5 in Treg cells (*Fig. *3). STAT5, activated by proliferation factor IL2, contributes to differentiation of Th1 and Th2 cells 95,96. Importance of IL6-induced STAT3 in Th2 cell differentiation has also been reported 97. STATs provide lineage specificity by promoting the differentiation of a given Th-cell subset while opposing the differentiation to alternative Th-cell subsets. For example, STAT4 promotes Th1 differentiation while inhibiting Th2 differentiation. On the contrary, STAT6 drives Th2 cell differentiation and inhibits Th1 cell specification 8. STAT5 in turn is a positive regulator of Th1, Th2, and Treg cell differentiation, while it negatively regulates differentiation of Th17 and Tfh cells 98–100. STAT3 promotes Th17 differentiation, but represses development of iTreg cells 101. Thus, STAT5 and STAT3 have opposing roles in regulating Th17 and Treg cells. Thus, STATs suppress the cell from differentiating to other T-cell lineages by directly competing with activators and recruiting other factors promoting the expression of repressive TFs.

## Lineage-specific TFs

There is a subset of TFs frequently referred to as ‘master regulators’, thought to be essential and sufficient for driving specific cell fates. Although the concept of master regulator TFs is useful in defining the key regulators, it undermines the complexity of Th-cell regulation and specification and is far from reality. It is becoming clear that interactions and integrated networks of regulatory TFs contribute to the differentiation program. To further understand the molecular mechanisms resulting in cell specification, system-wide approaches have been exploited to construct and decode the gene regulatory networks of TFs co-expressed within the cell 23,102. Functional genomics approaches along with high-throughput microarray and sequencing technologies have been instrumental for constructing TF-mediated transcriptional regulatory networks of several cellular differentiation and developmental programs, including those in T cells.

## Transcriptional control of Th1 cell differentiation

IL12 initiates Th1 cell differentiation by inducing signaling through binding to its receptor on activated CD4^+^ T cells which results in the phosphorylation and dimerization of STAT4. Phosphorylated STAT4 dimer translocates to the nucleus and binds to the DNA-binding sites in the regulatory elements of target genes, including *IL18RAP, IRF1, FURIN*, and *IFN*γ. IFNγ induces phosphorylation of STAT1, which activates the transcription of Th1-specific genes, *IL12R*β and *T-bet* (*TBX21*) that in turn act in a positive feedback loop to amplify the Th1 differentiation. STAT4 further promotes specification in Th1 cell lineage by negatively regulating the genes favoring Th2 cell differentiation 103–105. Thus, STAT4 and STAT1 initiate the Th1 lineage specification through binding to several *cis*-regulatory elements in the genetic loci for lineage-specific TFs and cytokines regulating their expression. Hence, they are regarded as lineage-specific pioneer factors as they initiate lineage specification in Th1 cells. In addition, STAT1 and STAT4 have been shown to be involved in establishing chromatin landscape in differentiating Th1 cells 10,106. IL2-induced STAT5 has also been shown to favor Th1 differentiation 107. T-bet is a lineage-specific TF driving Th1 differentiation. It stimulates transcription of lineage-specific cytokine gene *Ifn*γ by establishing a positive feedback loop and further potentiates the IL12 signaling by stimulating the transcription of the *Il12r*β*2* gene 108,109. However, STAT4 is required for T-bet to achieve IL12-dependent specification of Th1 cell lineage 110. Moreover, T-bet interacts with other transcriptional regulators of Th-cell differentiation, for instance with the members of Ets, and Hlx families, RUNX3 and BCL6, to oppose the alternative cell lineages by negatively regulating the expression of their lineage defining genes 111,112. T-bet physically interacts with BCL6 in Th1 cells to repress the transcription of genes favoring the alternative Th-cell lineages. At the later stage of Th1 cell differentiation, T-bet–BCL6 complex represses *Ifnγ* transcription to keep the production of IFN-γ in control as excessive production of IFN-γ could cause autoimmunity 113. RUNX3 physically interacts with T-bet to activate *Ifnγ* transcription by binding to its promoter and inhibits transcription of *Il4* cytokine by binding to its silencer region 111. Interestingly it was recently reported that T-bet and RUNX (RUNX1 and RUNX3) are also needed for *Ifnγ* transcription in IFNγ-producing Th17 Cells 114. Moreover, T-bet interacts with GATA3 (GATA-binding protein 3) to inhibit transcription of Th2 cytokine genes and block Th2 development 115,116. In addition, recent genome-wide studies have revealed that T-bet and GATA3 regulate the fate of the alternative cell lineages through a shared set of target genes 117,118. T-bet also blocks the differentiation of Th17 cell lineage by inhibiting RUNX1-mediated activation of RORC, a master regulator of Th17 differentiation 119,120. A recent study showed that T-bet inhibits the interferon regulatory factor 4 (IRF4) expression to repress Th17 cell lineage 121. Several other TFs have also been shown to regulate Th1 differentiation. TFs, ATF2, and ATF3 were reported to bind at *IFN*γ promoter and positively regulate the expression of *IFN*γ 122,123. In addition, we have shown that the *PIM* kinase family genes are induced by Th1-polarizing cytokines, indicating their role in regulation of Th1 cell differentiation 124. Further we have shown that PIM kinases promote Th1 differentiation by upregulating both *IFN*γ/*T-BET* and *IL-12*/*STAT4* pathways 125.

## Transcriptional control of Th2 cell differentiation

Combined with TCR-induced signals, IL4 initiates Th2 cell differentiation by phosphorylating STAT6, which then translocates to the nucleus and activates transcription of its target genes. These include *Il4* and *Gata3* genes, the key cytokine and TF, respectively, needed for Th2 cell lineage specification.

STAT6 is essential for Th2 differentiation as its genetic deletion severely hampers Th2 cell differentiation 126. STAT6 enforces GATA3 expression by exchanging the PcG complex with the TrxG complex at the genetic locus of *Gata3* during Th2 cell differentiation 127. Depletion of STAT6 using genetic deletion or RNAi and combined with global mapping of STAT6-binding sites both in mouse and human identified a large number of genes regulated by STAT6 during Th2 cell differentiation 81,82. In fact, in human up to 80% of genes differentially regulated during the early stage of Th2 differentiation were found to be regulated by STAT6 75. Aside from their role as regulators of transcription, STAT6 and STAT4 are important for setting up lineage-specific enhancer sites that control large number of genes in Th2 and Th1 cells, respectively 10. We have shown that STAT6 occupies the enhancer sites even before they become active during early stages of human Th2 cell differentiation 11. Therefore, environmental cytokine sensing STATs in combination with TCR-induced factors, such as NFAT and AP1, are involved in shaping the global chromatin landscapes that are utilized by lineage-specific TFs to drive chromatin remodeling and lineage-specific gene expression in respective Th-cell subsets. STAT6-independent pathways for Th2 differentiation have also been reported 128,129. Moreover, IL2-induced STAT5 is important for Th2 cell differentiation 8,130. STAT5 and GATA3 bind to the different *cis*-regulatory sites at the *Il4* locus to boost IL4 production in Th2 cells 131. In Th2 cells, global mapping of STAT3 binding revealed that STAT3 shares several binding sites at the regulatory sites of the target genes with STAT6 in differentiating Th2 cells 97. Therefore besides STAT6, both STAT3 and STAT5 are involved in positively or negatively regulating Th2 cell differentiation.

GATA3 is a lineage-specific key regulator of Th2 cell differentiation that auto-regulates its own expression by binding to its regulatory elements to further amplify Th2 differentiation. Genetic deletion of *Gata3* completely abolishes Th2 differentiation both *in vitro* and *in vivo* 132. On the other hand, enforced expression of GATA3 promotes Th2 differentiation by enhancing the expression of *Il5* and *Il13* genes 132. GATA3 promotes Th2 differentiation and maintains the cellular identity through distinct mechanisms—GATA3 induces transcription of Th2-specific cytokine genes (*Il4*, *Il5*, and *Il13* genes) itself through interacting with co-factors, and by inducing epigenetic modifications 133,134. Recent reports on genome-wide mapping of GATA3-binding sites suggested that GATA3 directly controls the expression of a large number of genes involved in Th2 differentiation 135,136. In addition, analysis of GATA3 binding from 10 developmental and effector T-cell lineages has revealed lineage specific as well as shared binding sites of GATA3 among different T cells. Binding of GATA3 to shared binding sites in distinct T-cell subsets suggests that cofactors binding along with GATA3 are important for determining the lineage specificity. 136. For instance, GATA3 cooperates with STAT6 for its binding to regulatory sites of its target genes in Th2 cells 135. GATA3 also acts as repressor of transcription of genes important for lineage specification and commitment of the alternative Th-cell lineages 117. For example, physical interaction of GATA3 with T-bet leads to repression of Th1 differentiation by inhibiting the transcription of *Il12r*β*2* and *Stat4* genes 115,117. Moreover, GATA3 also interacts with RUNX3 to suppress Th1 differentiation. RUNX3 in turn cooperates with T-bet for binding the *Ifnγ* promoter and *Il4* silencer regions to induce IFN-γ production, and suppress IL4 production 137. Recently, GATA3 was shown to interact with RuvB-like protein 2 (Ruvbl2) to facilitate the proliferation of Th2 cells through suppressing the expression of a CDK inhibitor, cyclin-dependent kinase inhibitor 2c (Cdkn2c) which is a critical regulator of cell cycle 138. Furthermore, GATA3 mediates remodeling of chromatin structure in the Th2 cytokine gene loci of *Il4*, *Il13*, and *Il5*. Overexpression of GATA3 in Th1 cells induced DHS at these Th2-specific cytokine gene loci. GATA3 interacts with co-activators or co-repressors to activate or repress the cytokine gene loci 128. For example, GATA3 interacts with Chd complex (a key component of NuRD chromatin remodeling complex) and HATs to mediate chromatin remodeling at Th2 cytokine locus, and its interaction with histone deacetylase inhibits the expression of *T-bet* 128. Recently, GATA3 was shown to inhibit *Ifnγ* expression in Th2 cells by recruiting EZH2 (H3K27me3 methyltransferase) to the *Ifnγ* locus 139. Taken together, GATA3 can mediate chromatin remodeling at a given gene locus to activate or repress gene expression.

Aside from STAT6 and GATA3, several other TFs contribute to the regulation of Th2 differentiation. For example, TFs, JUNB, and c-MAF cooperate to selectively activate the transcription of *Il4*, *Il5*, and *Il13* genes 140,141. In Th2 cells, IRF4 and NFATc2 complex bind to the *Il4* promoter and activate the transcription of *Il4* gene 142. STAT6-induced Gfi-1 TF selectively induces the expression of *Gata3* and endorses Th2 cell expansion 143. NOTCH TF binds to the *Gata3* promoter and the HSV enhancer of *Il4* and regulates the expression of *Gata3* and *Il4* in Th2 cells 144–146. TF Dec2 stimulates the expression of GATA3 and JUNB to favor Th2 differentiation 147. Moreover, interactions of SATB1 and TCF-1 with β-catenin regulate Th2 differentiation. Previous studies have shown that the expression of SATB1 is upregulated in Th2 cells 77,148. SATB1 regulates Th2 differentiation by employing β-catenin to the gata3 promoter and enhances the expression of *Gata3* and *Il5* 149,150. Alternatively, SATB1 supports Th2 cell differentiation by recruiting Ikaros TF to the gene locus of *T-bet* and *Ifnγ* to inhibit their expression 151. Likewise, TCF1 cooperates with β-catenin to enhance the expression of *Gata3* and inhibits the expression of *Ifnγ* gene, thus promoting Th2 differentiation 152. A recent study showed that TF YY1 regulates Th2 cell differentiation 153. It cooperates with GATA3 for binding to the Th2 cytokine loci and LCR in a Th2-specific manner. Furthermore, we have discovered a novel pathway of regulating Th2 differentiation through a protein of relevant evolutionary and lymphoid interest (PRELI). We found that PRELI downregulates Th2 cell differentiation through STAT6 154. Thus, these studies have established that multiple TFs are engaged in regulating Th2 differentiation.

## Transcriptional control of Th17 cell differentiation

Combined with TCR stimulation, Th17 cell differentiation can be induced by different combinations of cytokines both in human and mouse 79,155–158. TCR and cytokine stimulation induce a range of pioneer and lineage-specific and other TFs that control the specification and commitment of developing Th17. At early stages of Th17 differentiation, STAT3 initiates lineage specification by directly regulating the transcription of several target genes required for Th17 development including lineage-specific TFs, RORα, and RORγt 99,159. Recent studies in mouse system indicate that STAT3 controls the expression of several target genes including key TFs, such as *Batf, Irf4, Ror*α*, Ror*γ*t, Runx1, Fosl2, Ahr,* and *c-Maf*, and key cytokines, such as *Il17*α, *Il17f*, and *Il21* 23,80. Our unpublished data in human Th17 cells are consistent with the findings in the mouse cells. As in mouse system, STAT3 controls the expression of large fraction of genes including key TFs such as *STAT1, BATF, IKZF3, RUNX1, FOSL2, BCL6, IRF9*, and its own expression in human Th17 cells. In addition, we discovered a large number of STAT3-bound genes which have not been previously reported in the mouse studies (Tripathi SK *et al*., unpublished data).

RORγt is the lineage-specific TF of Th17 cells. Enforced expression of RORγt in STAT3 knockout mice induces *Il17a* expression, while RORγt deficiency results in complete inhibition of Th17 differentiation demonstrating that RORγt is essential and sufficient for generation of Th17 cells 160–162. Several other TFs expressed in Th17 cells positively or negatively regulate Th17 cell differentiation. For example, TFs including RORa, RUNX1, BATF, JUN, IRF-4, AHR, NOTCH1, and c-MAF, Aiolos, Ikaros, IkappaBzeta, IKK α, and HIF1α promote Th17 differentiation through various signaling pathways 160,162–173. On the other hand, TFs that suppress Th17 development include T-bet, FOXP3, GF1, ETS1, TCF1, EGR2, Th-POK, Jagged-1-Hes-1, TWIST1, PPAR-γ, KLF4, ELF4, ID3, and IRF8 84,118,171–180,184. Taken together, several TFs are important for modulating Th17 differentiation program.

## Transcriptional control of T-follicular helper (Tfh) cell differentiation

Tfh cells are relatively new subtype of effector CD4^+^ T cells and named after their location at follicular zones of germinal centers and their ability to provide help for B cells 185,186. Gene expression profiles and functions of Tfh cells compared to other effector Th cells make them a functionally distinct Th-cell subtype 185. Differentiation of Tfh cells is induced by IL6 and IL21, and the cells express C-X-C type chemokine receptor, CXCR5 and lineage-specific TF BCL6 187–192. Depletion of BCL6 in CD4^+^ T cells results in a failure to produce Tfh cells, whereas BCL6 overexpression promotes Tfh cell development indicating that BCL6 is necessary and sufficient for Tfh cell differentiation 83,193,194. Furthermore, BCL6 is a transcriptional repressor acting on the transcription of lineage-specific TFs of alternative Th-cell lineages, such as *T-bet* (Th1), *Ror*γ*t* (Th17), and *Gata3* (Th2) 195. However, expression of BCL6 is not restricted to Tfh cells, but expressed in other Th lineages as well 196. A BCL6 repressor, B-lymphocyte-induced maturation protein-1 (BLIMP1) can directly negatively regulate BCL6 expression via binding to its *cis*-regulatory regions 197. Genetic deletion of *Blimp1* in CD4^+^ T cells potentiates Tfh differentiation while enforced expression of BLIMP1 inhibits the process 197. Other regulators of Tfh cell differentiation include STAT3/5, IRF4, c-MAF, and BATF. STAT3 depletion significantly reduced the CXCR5^+^ Tfh cells as well as caused defective germinal center responses and B cell help both in human and mouse 198,199. On the other hand, STAT5 negatively regulates Tfh cell development and function 100,200. c-MAF cooperates with BCL6 to induce the differentiation of Tfh cell lineage 201. BATF in turn cooperates with JUN to promote Tfh differentiation by positively regulating the expression of BCL6 and c-MAF via binding to their regulatory regions 202,203. IRF4 plays a critical role in the regulation of Tfh differentiation. In Tfh cells, IRF4 cooperates with STAT3 or BATF–JUN complex to regulate the expression of several genes including *Blimp-1*. Genetic deletion of *Irf4* results in reduced STAT3 binding and Tfh cell differentiation 204. Though, major regulators controlling Tfh cell differentiation are well defined, the gene regulatory network of these and many other TFs driving specification and commitment of Tfh cell deserve more attention in the future.

## Transcriptional control of Th9 and Th22 cell differentiation

Combination of TGF-β and IL4 cytokines coupled with TCR activation initiate Th9 cell differentiation in naive CD4^+^ T cells by inducing the expression of PU.1 (purine-rich box 1) and IRF4 directly regulating the transcription of the *Il9* gene through direct binding to its regulatory elements 172. In Th9 cells, IL4 activates *Stat6* and *Irf4* expression, while TGF-β stimulates the expression of *PU.1*. PU.1 is a major regulator of Th9 differentiation. PU.1 inhibits the transcription of Th1-specific *T-bet* and Th2-specific *Gata3* and induces IL9 expression 205. Enforced expression of *Pu.1* in CD4^+^ T cells greatly enhanced Th9 cell development by TGF-β and IL4, while deficiency of *Pu.1* aborted Th9 cell differentiation 206. Similar experimental approach revealed that IRF4 had a similar effect on Th9 development as PU.1 207. Both PU.1 and IRF4 bind to the *Il9* promoter to induce transcription of *Il9* gene 206,207. The role of other TFs (yet not completely studied) in Th9 differentiation have been suggested. Computational analysis of regulatory sites at *Il9* locus has identified binding sites for several other TFs, such as AP-1, NF-κB, NFAT, GATA3, GATA1, STATs, SMADs, and NOTCH 205. Moreover, recent studies have indicated the role of NF-κB, Notch receptors, BATF, and Smad2/Smad3 in regulating Th9 responses 208,209. However, the mechanisms by which these TFs regulate Th9 development remains to be further studied.

Like Th9, Th22 is also a relatively new subtype of CD4^+^ Th cells, characterized by the secretion of IL22 210–212. IL6 and TNF induce Th22 cell differentiation, and AhR is considered to be a key factor for Th22 development 210,213. The molecular mechanisms and transcriptional factors involved in differentiation of Th22 cells are poorly characterized and need to be further investigated.

## Transcriptional control of Treg cell differentiation

Treg cells are classified into two major sub-groups, i.e. natural Treg (nTreg) cells derived from thymus and extrathymically derived adaptive or induced Treg (iTreg) cells. nTreg cells express IL2Rα chain (CD25) and FOXP3 which are critical for their development and immunosuppressive activity 214. iTreg cells that can be induced from CD4^+^ T cells upon treatment with TGF-β also express FOXP3. FOXP3 is a lineage-specific transcriptional regulator important for the development and homeostasis of Treg cell. FOXP3 expression is required for Treg-mediated tolerance both in mice and human because *Foxp3*-deficient Treg cells have been linked with severe autoimmunity 215–217. Global mapping of FOXP3-binding sites in Treg cells revealed that FOXP3 is actually only partly accountable for Treg signatures 218–222 suggesting the role of other TFs in the regulation of Treg cell development 222,223. In fact, FOXP3 physically interacts with other nuclear factors to cooperate in determining the Treg signature and functions 224. Furthermore, it was recently shown that a number of TFs, such as EOS, IRF4, SATB1, LEF1, and GATA-1, can work together with FOXP3 to form a transcriptional network governing Treg cell differentiation 225. However, the role of TCR signaling induced TFs, such as NF-κB, NFAT, AP-1, and FOXO1 were shown to regulate development and function of Treg during early stages of differentiation 224. For instance, NF-κB cooperates with FOXP3 to regulate the gene expression program in effector Th cells 226. Upon TCR/CD28 stimulation, NF-κB family member, c-Rel, regulates *Foxp3* expression by direct binding to its regulatory DNA regions 227. NFAT also regulates the development of Treg cells either through physically interacting with FOXP3 228 or by directly binding to the *Foxp3* promoter 229. Likewise, AP-1 regulates FOXP3 expression by directly binding to the *Foxp3* promoter 229. CREB binds to the CNS2 element of *Foxp3* gene to regulate FOXP3 expression 224. Furthermore, FOXO family promotes differentiation and function of Treg cells via a different mechanism 230. EOS is a TF that regulates Treg cell differentiation by participating and assisting FOXP3-mediated gene repression 231. HELIOS TF modulates the function of Treg cells by mediating epigenetic silencing of *Il2* gene transcription 232. Furthermore, RUNX family members, such as RUNX1 and RUNX3 support the differentiation and function of Treg cells by regulating FOXP3 expression either by direct binding to *Foxp3* promoter or by physically interacting with FOXP3 233,234. Both *in vivo* and *in vitro* studies have shown that STAT5 endorses Treg differentiation through different mechanisms 235,236.

## Epigenetic control of lineage specification and commitment in Th cells

Aside from the TF-mediated transcriptional networks and signaling pathways, various epigenetic mechanisms, such as DNA methylation, histone modifications, chromatin remodeling complexes, and ncRNA play a crucial role in driving gene expression programs as shown in various cellular differentiation systems 29,31,32,47,73,237 (*Fig. *1). In the pluripotent and multipotent progenitor cells, most of the developmental genes are inactivated or expressed at very low levels and many of them have so called bivalent chromatin structures 49. Nevertheless, these bivalent chromatin structures change into monovalent active or repressed structures causing either activation or repression of gene expression. These observations suggest that chromatin modifications can regulate gene expression and contribute to cell fate during development 49. Epigenetic changes in the chromatin structures are directed by different epigenetic modifying factors. For example, in ES cells, the regulatory DNA regions of pluripotency genes, such as OCT4 or NANOG, are marked with distinct histone modifications such as H3K4me3 on promoters and H3K4me1 on enhancers 29,52,56,57,238. Likewise, CD4^+^ Th precursor cells can develop into distinct subsets (*Fig. *3). Various layers of epigenetic control have been suggested to play a role in determining the lineage specification and commitment programs of a given CD4^+^ Th-cell fate detected by the expression of lineage-specific TFs and cytokines. In naive CD4^+^ T cells, the gene loci of these TFs and cytokines are inactive or expressed at a low level and marked by repressive monovalent or bivalent chromatin marks, respectively. Upon differentiation, repressive monovalent structures are erased or active monovalent structures are gained hence permitting the transcription 6,239. Genome-wide mapping has revealed the profiles of the epigenetic modifications, such as DNA methylation, histone modifications, DHS, and ncRNAs in Th cells 10,47,82,240–244. The results obtained from these studies have enhanced our understanding of the epigenetic mechanisms regulating Th-cell development and commitment. Furthermore, studies have shown that ncRNAs appear to be important in determining lineage-specific gene expression signatures in differentiating Th cells 242. Interestingly, recent studies have linked epigenetic regulation with disease states originated due to uncontrolled Th-cell activity 11,23,245,246. Moreover, comparison of epigenetic status of various cells of the immune system in patients with autoimmune diseases with healthy controls revealed differences in the chromatin state at loci for key genes and pathways. These studies have provided new insights that could help to better understand the pathogenesis of autoimmune diseases as well as lead to the foundation for developing epigenetic biomarkers for disease activity in immune-mediated diseases.^247^–^249^.

## Epigenetic control of Th1 and Th2 differentiation

The initial confirmation of the role of epigenetic mechanisms in regulating Th cells resulted from studies with DNA methylation inhibitor, 5-azacytidine. Treatment with this compound caused an increase in the secretion of IL2 and IFNγ cytokines in Th1 cells, while treatment with HDAC inhibitors resulted in enhanced secretion of IL4 cytokine in Th2 cells 250. These findings were further complemented by studies on genetic deletion of *Dnmt1* and *Mbd2* genes. These proteins mediate gene silencing through recruitment of HDACs and chromatin remodeling complexes to the DNA methylation sites. *Dnmt1* and *Mbd2* knockout mice had increased transcription of *Ifn*γ and *Il4* genes in Th1 and Th2 cells, respectively. These cells also lost the ability to repress the transcription of cytokine gene associated with an alternative lineage indicating the potential role of epigenetic mechanisms in the regulation of Th1 and Th2 cell lineage specification and commitment 251. Furthermore, in Th1 cells, a chromatin remodeling complex gene, Brahma related gene 1 (BRG1), is a component of STAT4-associated chromatin remodeling complex that mediates nucleosome positioning and chromatin remodeling at *Ifn*γ promoter and induces transcription of *Ifn*γ gene 252. Moreover, *Mll* gene (a histone methyltransferase) deletion caused reduced expression of Th2-specific cytokines *Il4* and *Il13* in memory Th2 cells indicating its role in the maintenance of the expression of these cytokines 253. Another *Mel18* (H3K27me3 binding poly comb repressor complex 1 protein) gene knockout caused inhibition of *Gata3* gene expression in Th2 cells 254. Recently, a methyl transferase SUV39H1-mediated methylation of lysine 9 of histone 3 (H3K9), was reported to associate with repressive HP1 protein to maintain the transcriptional silencing of Th1 gene loci, hence providing stability to Th2 cells 255.

Until recently, most studies on the epigenetic mechanisms in Th cells were concentrated on revealing the changes associated with the chromatin structures and accessibility at cytokine gene loci in Th1 and Th2 cells. These cytokine gene loci are regulated via their *cis*-regulatory elements, including promoters, enhancers, silencers, and insulators. The epigenetic mechanisms controlling gene expression patterns through these cytokine loci are discussed in detail elsewhere 13,239. Naive CD4^+^ T cells express low levels of the *T-bet* and *Gata3* gene, and cytokine genes, *Ifn*γ*, Il4*, and *Il13* 256. Chromatin state at the genetic loci of these TFs and cytokines are either inactive or in a poised state marked by low DHS, histone modifications, and a high degree of CpG methylation 253,257–259. On the other hand, in Th1 and Th2 cells, these gene loci are associated with gain of DHS, permissive histone modifications, and loss of repressive histone modifications as well as DNA methylation (DNA demethylation) to maintain active gene repertoire of lineage-specific TFs and cytokines for specific cell lineages 257–260. Thus, while gene loci for the lineage-specific TFs and cytokine genes are marked with permissive epigenetic state in a given Th-cell lineage, repressive epigenetic states take place in the opposing cell lineages.

Advancements of high-throughput sequencing technologies have enabled us to generate genome-wide map of DHS, nucleosome positioning, histone modifications, and DNA methylation to reveal the global epigenetic states associated with naive and differentiated Th cells, both in human and mouse 10,23,47,82,240,243,244,261. These studies revealed that changes in chromatin states due to distinct epigenetic modifications are directly correlated with gene transcription in T cells. Permissive histone modifications, such as H3K4me1, H3K4me2, H3K4me3, H3K9me1, H4K20me1, H3K79me3, H3K27me1, and H3K27ac are associated with active gene transcription, while repressive histone modifications, including H3K9me3, H3K27me2, and H3K27me3 are associated with gene repression. As mentioned earlier co-localization H3K27me3 and H3K4me3 at promoters form a bivalent domain associated with low gene expression state 49,244,262. For example, *T-bet* and *Gata3* gene promoters are marked with bivalent domains with low expression in naive T cells and are hence poised for expression or repression in differentiating T-helper cell subsets 244.

We recently performed global chromatin state analysis at 72 h of Th1 and Th2 differentiation that identified thousands of lineage-specific enhancers. Our analysis revealed that even at this early stage of differentiation process, enhancer-specific gene regulation is at work in determining the fate of developing cell lineages 11. DHS reflects an open chromatin structure and is presumed to be active chromatin. Overlap of publically available DHS data from the ENCODE consortium for Th1 and Th2 cells after 7 days of polarization with our enhancer analysis revealed the fate of identified lineage-specific enhancers during the course of Th1 and Th2 cell differentiation 240,263. We observed that 30% of Th1 and Th2 cell-specific enhancers were active (marked with both H3K4me1 and H3K27ac) at 72 h. Based on DHS data, of these active cell-specific enhancers around 76% of the Th1-specific and 99% of the Th2-specific enhancers remained active at 7–10 days of Th1 or Th2 differentiation, when most of the cells are fully committed. This observation suggests that these enhancers were needed for the maintenance of cell fate commitment throughout the differentiation process. However, those enhancers that were no longer hypersensitive reflect enhancers important for driving early lineage specification. In our analysis, enhancer elements marked with H3K4me1 but lacking H3K27ac were termed as ‘poised enhancers’. Of the poised enhancers, 21% in Th1 and 78% in Th2 cells gained DHS at later stage suggesting that these enhancers become active in cells committed to their respective lineages. This suggests that the epigenetic status is established before the commitment of cell fate 11. Further TF motif analysis of these lineage-specific enhancers revealed the binding of lineage-specific TFs. For example, Th1 lineage-specific enhancers were enriched with TF motifs for key TFs STAT4, ATF3, STAT1, and JUN. Th2 lineage-specific enhancers in turn had binding sites for key TFs including STAT6, GATA3, GFI1, NFIL3, and PPARG. Using a subset of Th2-specific enhancers, we showed that STAT6 binding takes place even before enhancers become active. Other studies indicate that STATs play a crucial role in setting up the chromatin landscapes during Th1 and Th2 cell differentiation 10,73. These studies have revealed that STATs control global enhancer marking in Th1 and Th2 cells as genetic deletion of *Stat* genes resulted in loss of large number of lineage-specific enhancers based on H3K4me1 and P300 ChIP-seq data.

## Epigenetic control of Th17 and Treg differentiation

Previous studies have revealed that genetic loci of Th17 lineage-specific cytokine genes, *Il17a* and *Il17f* are associated with permissive histone modifications like H3K27ac and H3K4me3 in Th17 cells and are regulated by lineage-specific pioneer TF STAT3 264,265. A CNS (CNS2) regulatory region upstream of *Il17a* cytokine locus has binding sites for Th17 lineage-specific TF RORγ 162. In addition, IKKα (inhibitor of nuclear factor-κB kinase-α) is required for phosphorylation of histone H3 at *Il17a* locus to activate *Il17a* gene expression and it drives the commitment to Th17 cell lineage 266. The genetic loci for key Th17-specific TFs and cytokine genes are epigenetically instable in Th17 cells, thus revealing the plastic nature of Th17 cells. Therefore, cell-extrinsic factors can modulate the fate of Th17 cells 267. Moreover, global analysis of histone modifications in Th17 cells revealed that promoters of cytokines genes, such as *Il21*, *Il17a*, *Il17f*, *Il1r1*, *Il17re*, as well as lineage-specific *Ror*γ*t* were enriched with active H3K4me3, correlating with their expression pattern. In contrast, enrichment of repressive H3K27me3 mark on *Il17*, *Il21*, and *Ror*γ*t* promoters was identified in alternative Th-cell lineages. Interestingly, *Gata3* and *T-bet* gene promoters were enriched with bivalent domains, suggesting that these cells are poised for development toward Th1 and Th2 cells 244. Thus, this study revealed differences in the epigenetic profiles that correlate with selective gene expression profiles. Further studies indicated that cell-specific TFs, such as STAT3, IRF4, AP1/BATF, and RORC modulate DNA accessibility at genetic loci of Th17 genes, such as *Il17a, Il17f, Il23r, Ccl20, Il1r1,* and *Ltb4r1* by regulating histone modification status 23,80. Moreover, using a pharmacological inhibitor of BET function, BET family of proteins was shown to modulate Th1 and Th17 responses by regulating chromatin structure and gene transcription through binding acetylated lysine residues in histones 268,269. Even though these and other studies have begun to highlight the epigenetic control of lineage specification and function in immune cells 270,271, how these changes are incorporated is far from understood.

FOXP3-expressing nTreg and iTreg can suppress function of effector Th-cell subsets 222,272,273. The differences in reprogramming tendency of nTreg and iTreg cells is due to their epigenetic status which is complemented with the histone modification and DNA methylation states of the *Foxp3* and *Rory* locus, respectively. iTreg cells are positive for *Rory* expression while *Il17a* expression is repressed due to the presence of permissive H3K4me3 mark at *Rory* locus and repressive H3K27me3 mark at *Il17a* locus. However, in nTreg cells, both *Rory* and *Il17a* genes are repressed and their promoters are marked by H3K27me3 244,274. *Foxp3* locus is methylated in naive and stimulated CD4^+^ T cells, as well as in iTregs, but the locus is demethylated in nTregs. DNA methylation process at the *Foxp3* locus is directed by the DNA methyltransferases, DNMT1 and DNMT3b 275–277. Further, inhibitors of DNMTs result in increased number of FOXP3-expressing Treg cells 278. Global analysis of DNA methylation landscape in CD4^+^CD25^−^ conventional T (Tconv) cells and CD4^+^CD25^high^ Treg cells showed that a Treg-specific DNA hypomethylation correlated with the expression of genes vital for Treg cell function, such as *Foxp3, Ctla4, Il2ra, Cd40lg, Ikzf2 (Helios), Ikzf4 (Eos), and Tnfrsf18 (GITR)* 277,279. In contrast, global analysis of H3K4me1 and H3K4me3 maps in human Treg cells revealed lineage-specific histone methylation patterns 48. This study showed that proximal promoters of *CTLA4, IL2RA*, and *TNFRSF18* were marked with active H3K4me3 modifications both in CD4^+^CD25^+^FOXP3^+^ Treg and activated conventional CD4^+^CD25^+^FOXP3^−^ T cells. Non-promoter distal regions in turn were enriched with H3K4me1 enhancer mark and displayed a high degree of lineage specificity in binding pattern for Treg-specific genes, including *IL2RA, FOXP3, CTLA4*, *and TNFRSF18*. These results support vital function of enhancer elements in marking lineage-specific gene expression programs in Treg cells, which is consistent with observations drawn from studies on other cell systems 11,46,52.

## Role of ncRNAs in Th-cell differentiation and development

The role of ncRNAs in determining lineage specification and commitment during Th-cell differentiation and development is poorly defined. However, initial studies showed that depletion of microRNA-processing endonucleases *Drosha* and *Dicer* genes caused disturbances in microRNA-processing that generate miRNAs important for the stability and function of Th cells, indicating the role of regulatory ncRNAs in T-cell differentiation and associated immune-mediated diseases 241,242,274. Efforts have been made to build ‘microRNome’ or ‘lncRNome’ to categorize a set of microRNAs and lncRNAs regulating lineage commitment during Th-cell differentiation both in mouse and human lymphocytes 75,280. Furthermore, global analysis of miRNAs identified several lineage-specific miRNAs in nearly 50 immune cell types, suggesting their roles in determining lineage specificity 281. Several other studies have focused on identifying unique miRNAs that regulate the development and function of the Th cells. For instance, miR-125b maintains the naive state of precursor Th cells, miR-182 promotes clonal expansion, miR-326 encourages Th17 development, and miR-146a endorses suppressive function of Treg cell lineage 242,282–284. In addition, miRNA-155 controls Treg and Th17 cell development 280, miR-10a inhibits *BCL6* expression and regulates the flexibility of Th cells 285, and miR-17–92 cluster controls Th1 cell differentiation 286. Moreover, recent studies showed that miRNAs miR-21, miR-301a, and miR-146b regulate Th-17 differentiation 188,287. A very recent study demonstrated that miR-210 regulates Th17 cell differentiation through modulating the expression of HIF1-α 288.

lncRNAs that are regulated during normal and disease states, use a range of different molecular mechanisms to modulate gene expression. Several lncRNAs have been associated with T-cell differentiation and function. For instance, NRON lncRNA regulates NFAT function 289, lncRNA, GAS5 halts T-cell growth 290, and lncRNA from the T early α promoter (TEA) and NeST lncRNA [also called Theiler's murine encephalitis virus possible gene 1 (TMEVPG1)] is selectively expressed in Th1 cells and drives *Ifn*γ expression 291–293. Global analysis of lncRNAs in mouse CD8^+^ T cells identified several lncRNAs potentially regulating lymphocyte activation and differentiation 294. In addition, in mouse system, a recent genome-wide profiling of lincRNAs from distinct T-cell lineages revealed various lincRNAs with lineage-specific expression profiles 75. Most of our current knowledge on lncRNA and miRNA function in Th cells comes from studies done in mouse and studies in human deserve further attention. Combination of distinct genome-wide datasets with disease-associated SNPs or directly identifying lncRNAs in samples from patients with diseases will help in identifying mutations associated with altered ncRNA expression, their correlation with disease states, and hopefully also provide mechanistic understanding about their functions.

## TFs in shaping the epigenetic landscape in differentiating Th cells

**Fig 4:**
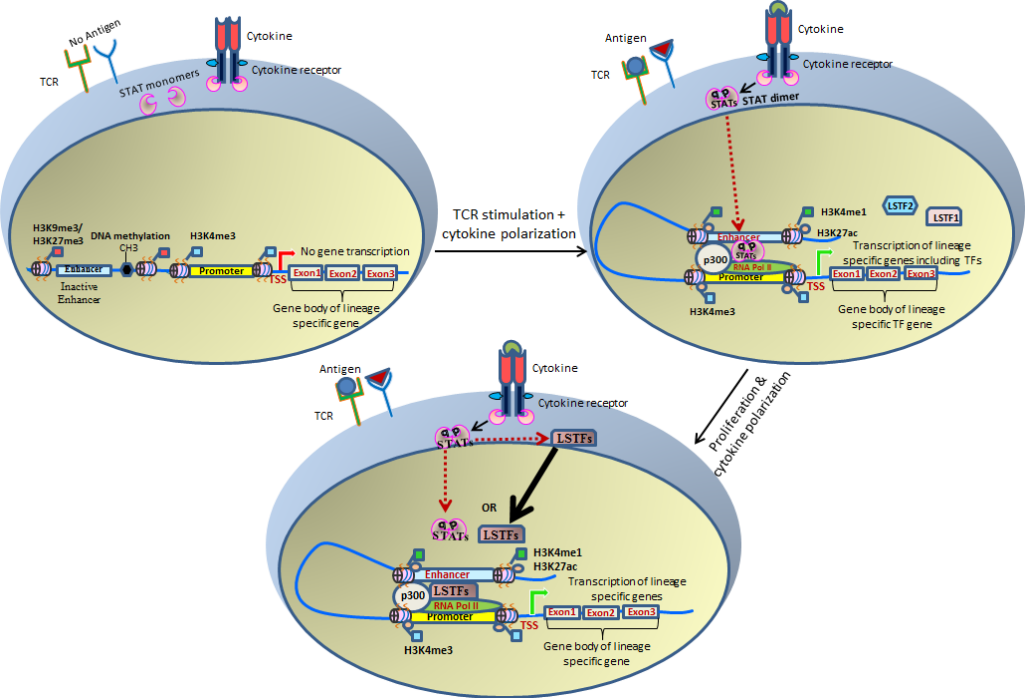
STATs establish enhancer sites to induce enhancer-mediated regulation of lineage-specific gene expression program. Combined activation of TCR and cytokine receptors convert inactive closed chromatin state to active open chromatin structure by removing inactive epigenetic marks and adding active epigenetic marks. The chromatin landscape created by STATs is further utilized by LSFs to mediate lineage-specific gene expression program.

Genome-wide studies have identified targets of STATs shaping the gene expression programs to promote differentiation of specific Th cell-lineages while opposing alternative fates 23,80–82. These studies have revealed that around 20% of the STAT-binding sites are in the promoters, whereas over 70% of them are in the intergenic and intronic regions. These results suggest that STATs utilize these *cis*-regulatory elements to regulate gene expression in relevant Th subsets. It remains an open question what are the factor(s) involved in establishing the lineage-specific epigenetic status in Th cells. As epigenetic status of a cell can be changed in response to environmental factors (for example stimulation with environmental cytokine milieu), STATs as sensors of environmental cytokines and amplifiers of the Th-cell differentiation are candidates for shaping the chromatin landscape in differentiating Th cells. Recent studies suggest that aside from directing transcription through binding to *cis*-regulatory elements, STATs regulate epigenetic landscape by influencing the histone modification status of the cell at these regulatory regions 7,10,11,23,80,82. Further, genome-wide analysis revealed that STAT3 and STAT4 have influence on active promoter mark, H3K4me3 10,80,82. Conversely, STAT6 has been shown to affect H3K27me3 status 82. In the recent past, much of the focus has been on these distal regulatory enhancer elements in instructing lineage-specific gene expression programs in various cells including T cells 10,11,46,52. STAT1 and STAT4 participate in creating sites for enhancers in differentiating Th1 cells 106,295,296. STATs were also shown to alter the repressive histone modification state into active histone modification state at these enhancer sites 10. On the basis of ChIP analysis of subsets of Th2-specific enhancers identified from global analysis, we showed that these enhancers were already marked with H3K4me1 and STAT6 binding at early stage of differentiation while they gained H3K27ac at a later stage of Th2 development. This indicate that marking of enhancers and binding of STAT6 to these enhancers takes place first while they become fully active at the later stage differentiation 11. Thus, STATs may have function in shaping enhancer repertoire for lineage-specific TFs and enable them to bind at these enhancers to regulate expression of genes (*Fig. *4). Also, these studies have suggested that STATs play an important role in guiding lineage-specific gene expression programs through multiple mechanisms that cause changes in global gene transcription and histone epigenetic modifications.

## Inferring the significance of disease-associated SNPs in the gene regulation

A large number of SNPs has been associated with diseases and listed in GWAS. However, many of these studies have failed to correlate SNPs with functional disease phenotype, probably because many of these SNPs are present on non-coding regions. Thus, to determine how these SNPs can potentially regulate complex phenotypes relies on the activity of the regulatory elements harboring SNPs. Analysis of expression quantitative trait loci (eQTLs) revealed that regulatory SNPs (rSNPs) can change the expression of associated genes 297,298. According to Hindorff *et al*. 299, around 90% of disease-associated SNPs are enriched in the intronic and intergentic regions, respectively. ENCODE, NIH Road Map project, and other studies focusing on global assessment of epigenetic profiles have shown that disease-associated SNPs were enriched in the gene regulatory regions of genome, such as promoters and enhancers 23,240,300,301. Interestingly, most of the enhancers are located in introns or intergenic regions where there is also enrichment of SNPs suggesting that SNPs regulate the chromatin accessibility through these regulatory regions. A recent report 263, which compares the localization of disease-associated SNPs to DHS, revealed similar observations. Furthermore, many of these identified SNPs are not causative SNPs and thus identification of specific functional variants at individual GWAS loci remains challenging. While earlier studies have focused on studying the statistically enriched SNPs at any given locus (lead SNPs), several other SNPs are found in linkage disequilibrium (LD) with a lead SNP that enable them functional and trait associated.

**Fig 5:**
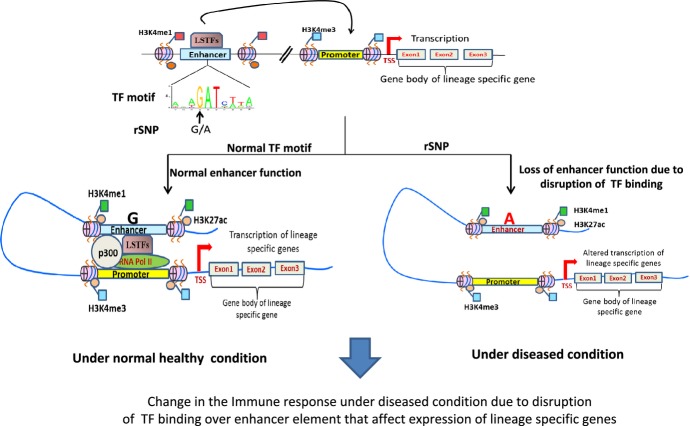
Regulatory single-nucleotide polymorphisms (SNPs) disrupt TF binding on a lineage-specific enhancer, which in turn results in loss of enhancer-mediated lineage-specific gene expression program. SNPs identified in genome-wide association studies (GWAS) were found to be greatly enriched in intergenic and intronic regions that are also likely sites for enhancer elements. SNPs can disrupt transcription factor (TF)-binding sites within enhancer regions. Here, we show a model where under normal state, TF binds to an enhancer element and allow binding for histone acetyl transferases, p300, and RNA polII, to initiate the transcription of target genes. A SNP localized in TF-binding site within the enhancer region can cause a disruption of TF binding and result in attenuation of recruitment of p300 and RNA polII to the enhancer and thereby lead to loss of enhancer-mediated cell-specific gene expression.

We and others have integrated global epigenetic modifications data from distinct Th-cell lineages with SNPs from public GWASs catalogs to determine whether these disease-associated SNPs are regulatory SNPs 11,23. The integrative analysis revealed that a number of SNPs were localized within the TF-binding motifs on *cis*-regulatory modules (CRMs), including enhancers and promoters. Further, we experimentally validated in Th cells a panel of these regulatory SNPs. Disruption of the TF-binding sites over these CRMs resulted in changes in TF binding suggesting their role in regulating gene expression 11. Thus, in Th cell context, SNPs within lineage-specific TFBS on CRMs could cause dysregulation of lineage defining genes potentially resulting in modulation in Th cell-mediated immune responses (*Fig. *5).

## Future prospects in Th-cell differentiation and development

Recent studies have extended our understanding on molecular mechanisms of lineage specification and commitment during differentiation and development of Th-cell lineages. The advancement and availability of novel experimental techniques and next generation sequencing technologies has enabled us to comprehend the complex cellular information at the level of -omes, such as the transcriptome, epigenome, proteome, miRNAome, and interactome. Integration of genome-wide transcriptomics and epigenomics data with data from GWASs on immune-mediated diseases is starting to provide new insights into the molecular mechanisms involved in pathophysiology of immune-mediated diseases associated with Th-cell lineages. Characterization of non-coding regions (including ncRNAs) of the genome and their association with disease-associated regulatory SNPs is likely to reveal new aspects of the regulation of immune response. Efforts will be made to systematically investigate the dynamic interactions among the genome, proteome, and epigenome to establish the complete ‘regulome’. Systems level understanding of specification and commitment during human Th-cell differentiation will be crucial for implications in human health and diseases.
